# Preparation and Characterization of Graphene-Nanosheet-Reinforced Ni-17Mo Alloy Composites for Advanced Nuclear Reactor Applications

**DOI:** 10.3390/ma18051061

**Published:** 2025-02-27

**Authors:** Xiaoxin Ge, Yanxin Jiang, Xu Yu, Guopeng Zhang, Yunjia Shi, Bin Cai, Qing Peng, Hai Huang

**Affiliations:** 1 Key Laboratory of Material Physics, Ministry of Education, School of Physics, Zhengzhou University, Zhengzhou 450001, China; 2 International Joint Laboratory for Integrated Circuits Design and Application, Ministry of Education, School of Physics, Zhengzhou University, Zhengzhou 450001, China; 3 State Key Laboratory of Nonlinear Mechanics, Institute of Mechanics, Chinese Academy of Sciences, Beijing 100190, China

**Keywords:** graphene–Ni-17Mo alloy composites, powder metallurgy, microstructure, mechanical properties, molten salt reactors

## Abstract

Molten salt reactors (MSRs) offer advantages such as enhanced safety, reduced nuclear waste, and cost effectiveness. However, the corrosive nature of fluoride-based molten salts challenges the longevity of structural materials. Ni-based alloys, like Hastelloy N, have shown resistance to fluoride salt corrosion but suffer from issues like helium embrittlement caused by neutron irradiation. To address these concerns, the incorporation of graphene (Gr) into Ni-based alloys is being explored. Gr’s superior mechanical properties and irradiation tolerance make it a promising reinforcement material. In this study, a Ni-17Mo alloy, a simplified model of Hastelloy N, was combined with reduced graphene oxide (RGO) using powder metallurgy. The effects of milling time and sintering temperature on the microstructure and mechanical properties were systematically studied. The results indicated that optimal sintering at 1100 °C enhanced tensile strength and ductility. Additionally, RGO incorporation improved the alloy’s strength but reduced its elongation. This research highlights the potential of Gr-reinforced Ni-based alloys for advanced MSR applications, offering insights into fabrication techniques and their impact on material properties.

## 1. Introduction

Molten salt reactors (MSRs) are among the six most advanced Gen-IV nuclear fission reactor technologies, attracting considerable attention due to their inherent safety features, use of anhydrous cooling systems, reduced nuclear waste production, and cost effectiveness [1,2]. Despite these advantages, the highly corrosive nature of fluoride-based molten salt coolants presents significant long-term challenges for structural materials [3]. Conventional reactor materials are inadequate for the extreme operational conditions in MSRs. Ni-based alloys, however, demonstrate exceptional mechanical properties and outstanding resistance to high-temperature corrosion [4]. Leveraging these properties, researchers at Oak Ridge National Laboratory developed the Hastelloy N alloy (i.e., Ni-17 wt.% Mo-7 wt.% Cr), which demonstrated superior resistance to fluoride salt corrosion in the experimental MSRs [5,6]. Nevertheless, its limitations have become increasingly evident during the transition to commercial and industrial applications. During reactor operation, neutron irradiation and the presence of fission products induce microstructural changes in the structural materials, compromising their mechanical integrity and corrosion resistance [7,8]. In addition, Hastelloy N undergoes neutron-induced transmutation reactions, generating helium atoms [9]. These helium atoms tend to accumulate at grain boundaries, leading to reduced ductility and causing helium embrittlement, which severely limits the alloy’s service life in reactor cores [9,10,11,12]. Consequently, it is imperative to develop new nickel-based alloys with enhanced corrosion resistance, mitigated susceptibility to helium embrittlement, and improved mechanical strength to meet the rigorous demands of future MSR advancements.

The development of irradiation-tolerant materials with self-healing capabilities increasingly focuses on leveraging grain boundaries and heterointerfaces, a growing consensus within the scientific community [13,14,15]. Graphene (Gr) stands out due to its exceptional electronic and thermal conductivity, coupled with outstanding mechanical properties such as a high Young’s modulus (~1 TPa) and intrinsic strength (~130 GPa) [16]. Functionalized Gr is typically synthesized by oxidizing graphite to form graphene oxide (GO) with oxygen-containing functional groups, enhancing its reactivity [17]. The reduction of GO removes these groups, producing reduced graphene oxide (RGO) with vacancy defects [15,18]. As a two-dimensional nanomaterial, Gr and its derivatives show significant promise as a reinforcing component in metals, endowing them with unique functional properties [19]. Recent studies have demonstrated the superior irradiation tolerance of various Gr–metal composites, largely attributed to their ultra-strong and stabilized interfaces [20,21]. For instance, Si et al. [22] reported that Gr/W nanolayers with reduced period thicknesses exhibit remarkable resistance to irradiation, effectively lowering helium bubble density. Similarly, Liu et al. [23] observed that Gr/Al composites outperform unreinforced matrices in reducing irradiation-induced hardening, enhancing elongation, minimizing lattice expansion, and exhibiting unique deformation mechanisms under irradiation. Huang et al. [15] highlighted the ability of the Gr/Ni interface to attract, absorb, and eliminate interstitials, vacancies, and helium atoms or clusters, significantly mitigating residual irradiation defects. These findings suggest that Gr/Ni composites hold strong potential as novel irradiation-tolerant materials for advanced nuclear reactors. Despite this promise, challenges persist in advancing Gr–metal composites as structural materials for MSRs. One major unresolved issue is the interaction between solute elements (e.g., Mo, Cr, and Fe) and Gr’s C atoms under high-temperature preparation or irradiation conditions, which may result in metal carbide formation [24]. The binding of these carbides to the Gr surface could alter the interfacial structure, potentially influencing the interfacial defect trapping efficiency.

To attain the desired properties in high-temperature composite fabrication, optimizing the process parameters that govern microstructure control is essential [25]. Powder metallurgy (PM) is widely acknowledged as the preferred method for producing Gr–metal composites [26]. In comparison to casting, PM provides superior control over the microstructure, facilitating a more homogeneous distribution of Gr within the composites [27]. The effectiveness of PM is influenced by several factors, including the duration of ball milling, sintering conditions, and the reinforcement content [28]. Research has demonstrated that the duration of ball milling significantly impacts the microstructural characteristics of the powders [29,30]. Furthermore, sintering parameters—such as temperature, dwell time, and atmosphere—are critical determinants of the composite materials’ overall performance [31,32]. Precisely optimizing these parameters, particularly sintering temperature and dwell time, is vital for achieving high density and enhanced mechanical properties in Gr–metal composites. For instance, sintering at excessively low temperatures can result in higher porosity, compromising material performance [33]. Moreover, due to the strong van der Waals forces and hydrophobic nature of Gr, its dispersion in solvents is challenging, leading to agglomeration [34]. In contrast, GO, known for its hydrophilicity, is commonly used in experiments as a substitute [14]. As an example, Zhang et al. [35] employed PM to produce reduced GO (RGO)/Ni composites, showing that, compared to pure Ni, the composites with 0.3 wt.% GO exhibited increases in yield strength, tensile strength, and fracture elongation by 16.3%, 34.3%, and 36%, respectively. Chen et al. [36] fabricated Cu-based composites reinforced with Gr nanosheets at varying concentrations and found that the addition of Gr notably improved the mechanical properties of Cu. However, with increasing Gr content, the strengthening effect initially enhanced and then declined. The maximum elastic modulus and hardness saw improvements of 65% and 75%, respectively, compared to pure Cu. Nonetheless, there remains a paucity of research on the preparation methods for Ni-based alloys incorporating Gr. Furthermore, studies examining the influence of varying Gr content on the microstructural evolution and mechanical properties of Ni-based materials, as well as the associated strengthening mechanisms, are notably lacking.

In this study, the Ni-17Mo alloy was selected as a simplified model for Hastelloy N to explore the fabrication of Gr-nanosheet-reinforced alloy composites using a modified powder metallurgy approach. First, the effects of milling time on the uniformity of powder mixing and particle size were systematically investigated. Then, once the optimal milling time was determined, the powders were sintered at 1000, 1050, and 1100 °C, followed by mechanical property testing of the samples at each temperature. Lastly, the composites were produced by incorporating 0.5 vol.% RGO, and the microstructural characteristics and properties of composites with varying RGO contents were analyzed. Note that this study primarily focuses on the preparation and characterization of the composites by optimizing key processing parameters. A more comprehensive structural analysis and performance assessment, including molten salt corrosion resistance and high-temperature irradiation testing, will be conducted in future research.

## 2. Experimental Procedures

### 2.1. Raw Powder Preparation

**Figure 1:**
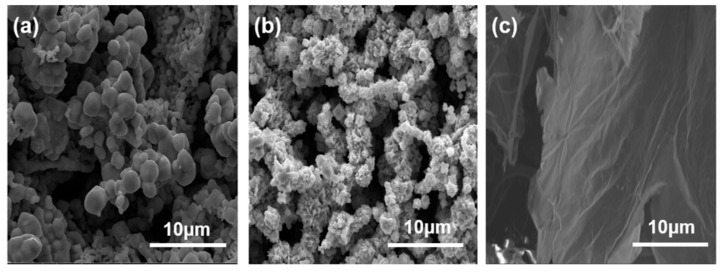
SEM images showing the morphology and particle size distribution of Ni powder (**a**), Mo powder (**b**), and GO powder (**c**) in their unprocessed forms.

The Ni powder (purity > 99.6%, Jiangyou Hebao Nanomaterials Co., LTD., Mianyang, China) and Mo powder (Changsha Tianjiu Metal Materials Co., LTD., Changsha, China), both produced via gas atomization, were selected as the raw materials (see Figure 1a,b). The GO, with a purity exceeding 99%, was synthesized using a modified Hummers method [15] and supplied by Nanjing XFnano Material Tech Co. LTD., Nanjing, China (see Figure 1c). A total of 100 g of spherical Ni-17Mo powder was placed into a ball mill tank, and steel balls were added at a ball-to-material mass ratio of 10:1. Argon gas was purged into the tank for 10 min before initiating the milling process. The planetary ball mill was operated at a speed of 300 rpm, with milling times set at 0.5, 1, 2, 4, and 8 h. To prevent excessive heat buildup that could adversely affect the powder properties, the milling process was paused for 5 min after every 0.5 h of operation. The mill’s rotation direction was reversed periodically to avoid powder adhering to the tank walls, which could reduce yield. After milling, the suspension was allowed to settle briefly, and the upper liquid was decanted. The residual powder was vacuum-dried at 60 °C for 10 h, resulting in mixed powder with a flake-like morphology. Furthermore, a suspension consisting of GO nanosheets was prepared through ultrasonic dispersion. This involved introducing a measured quantity of GO into deionized water, followed by sonication for 2 h to achieve a uniform suspension with a concentration of 1 g/L. The resulting GO suspension was transferred into a beaker and combined with Ni-17Mo powders that had been previously ball-milled in pure ethanol. The mixture was then subjected to mechanical stirring to ensure complete adsorption of the GO layers onto the flaky Ni-17Mo powder surfaces.

### 2.2. Synthesis of Alloy and Composite Bulk

**Figure 2:**
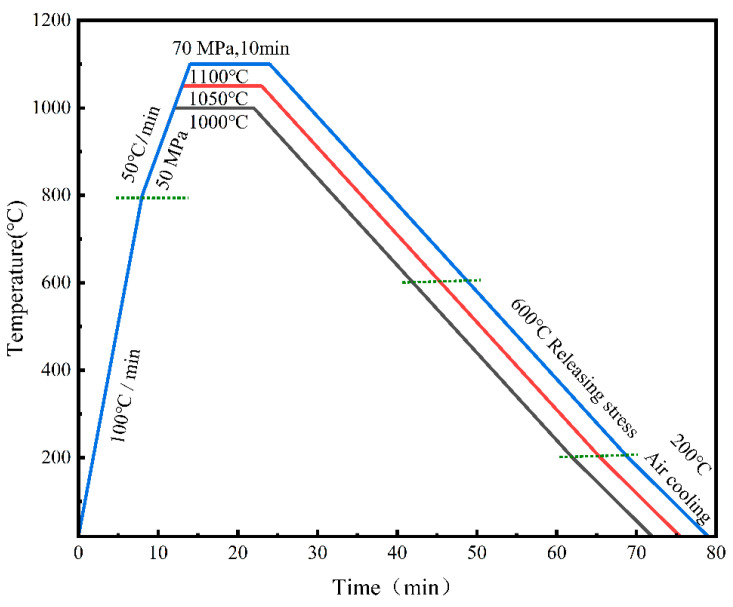
Schematic representation of the heating cycles used during the sintering of Ni-17Mo alloy powder compacts milled for 2 h at various temperatures, along with the processing route for composite powders. Note that dashed lines demarcate each sintering phase to distinguish stage-specific conditions.

The powder mixture was placed into a graphite mold with a 40 mm diameter. This mold was then positioned in a fast hot-pressing sintering furnace and subjected to sintering under a vacuum pressure of 10^−2^ bar. The sintering was conducted at temperatures of 1000, 1050, and 1100 °C for the Ni-17Mo alloy bulk and at 1100 °C for the 0.5 vol.% RGO/Ni-17Mo alloy composites. Note that this specific Gr concentration was not determined through optimization in this study but was selected based on extensive research indicating its effectiveness in enhancing material properties [35,37,38]. Its adoption aimed to assess the reliability of the proposed fabrication process. During the holding stage, a pressure of 70 MPa was applied for 10 min, with a heating rate of 100 °C/min and a cooling time of approximately 40 min. The final sintered sample had a diameter of 40 mm and a thickness of approximately 6 mm. Detailed information on the rates of pressurization, heating, and cooling throughout the sintering process is provided in Figure 2.

### 2.3. Microstructure Characterization and Property Measurements

The powder’s size, morphology, and microstructure were analyzed using two-beam scanning electron microscopy (SEM, Helions G4CX) and X-ray diffraction (XRD, PANalytical Empyrean, Almelo, The Netherlands) with a Cu-K_α1_ radiation source (λ = 1.5406 Å). SEM analysis was conducted in conjunction with energy-dispersive X-ray spectroscopy (EDS) at an accelerating voltage of 20 kV. XRD patterns were recorded over a 2θ range of 20–90° with a scan rate of 10°/min. The Raman spectra of the original, ultrasonically dispersed, and reduced GO powders were acquired using a Renishaw inVia Raman Microscope, Gloucestershire, UK. The measurements were conducted with a 532 nm He–Ne laser, a spectral resolution of 1 cm^−1^, and a 100× objective lens (numerical aperture: 0.85) utilizing the line-scan method. Additionally, the mechanical properties of the Ni-17Mo alloy at varying sintering temperatures and 0.5 vol.% RGO/Ni-17Mo alloy composites were evaluated using a universal testing machine (MTS-CMT5105, Eden Prairie, MN, USA) and a digital microhardness tester (HVS-50, Shanghai, China). Microhardness measurements were repeated at least five times with a 4.903 N load and 15 s dwell time. Uniaxial tensile tests were conducted at room temperature with a fixed crosshead speed of 2.0 mm/min.

## 3. Results and Discussion

### 3.1. Effect of Milling Time on Powder Mixture

**Figure 3:**
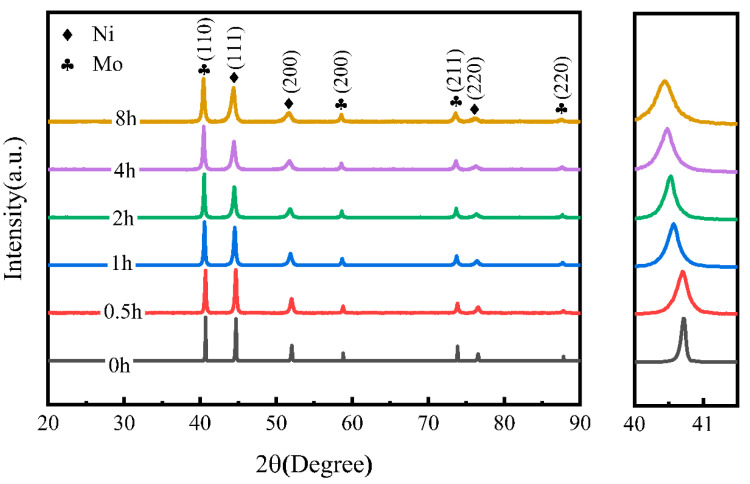
XRD patterns of Ni-17Mo alloy powders milled for different durations, illustrating the evolution of crystallographic phases with milling time.

Figure 3 presents the XRD spectrum of Ni-17Mo alloy powder, with milling times ranging from 0 to 8 h. Both the intensity and width of the diffraction peaks decrease and broaden with increasing milling duration. This trend suggests a reduction in the average grain size, accompanied by a rise in lattice distortion and crystal defects. A similar pattern has been reported in previous studies [39]. In addition, milling durations ranging from 0.5 to 8 h did not result in mechanical alloying of the elemental Ni and Mo powders.

**Figure 4:**
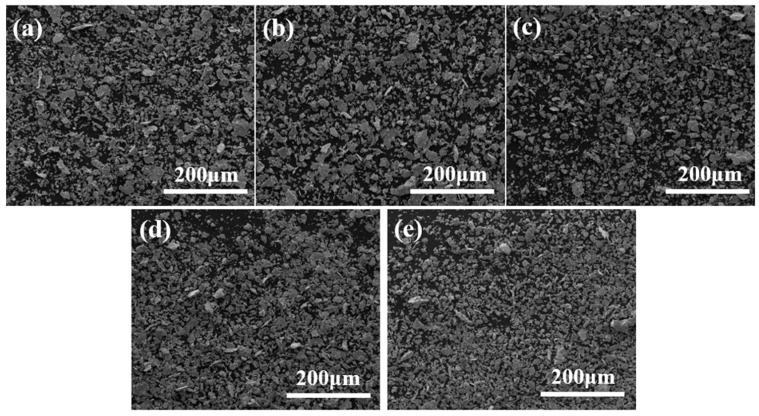
Low-magnification SEM images depicting the morphology and microstructural evolution of Ni-17Mo alloy powder particles after ball milling for 0.5 h (**a**), 1 h (**b**), 2 h (**c**), 4 h (**d**), and 8 h (**e**).

**Figure 5:**
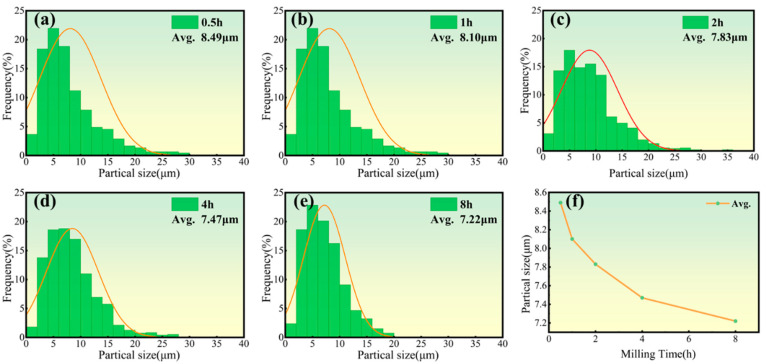
Particle size distribution curves (**a**–**e**) and their average sizes (**f**) for Ni-17Mo alloy powders milled over different durations ranging from 0.5 to 8 h, highlighting the effect of milling time on particle refinement and uniformity.

**Figure 6:**
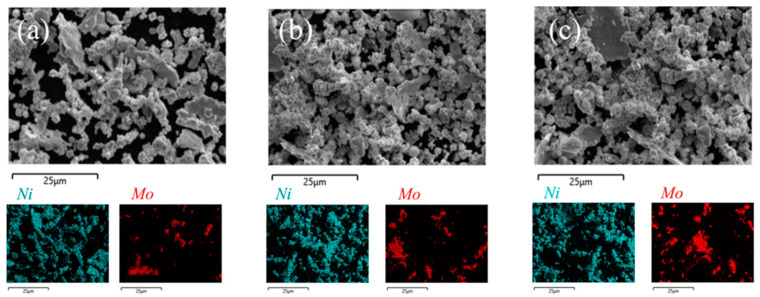
High-magnification SEM images of Ni-17Mo alloy powders milled for 0.5 h (**a**), 1 h (**b**), and 2 h (**c**), along with corresponding EDS elemental maps that illustrate the elemental distribution and microstructural features.

Figure 4 and Figure 5 illustrate the changes in the size and morphology of the alloy powder particles during ball milling. The average particle size was measured via SEM image analysis using software ImageJ 1.53t (National Institutes of Health, Bethesda, MD, USA), and the resulting size distribution was modeled with a log-normal function, a standard method for analyzing ball-milled powders [40]. Clearly, as milling time increases from 0 to 8 h, the average grain size slightly diminishes from 8.49 to 7.22 μm. Initially, both Ni and Mo powders were nearly spherical in shape. However, after ball milling for various durations, significant alterations in particle morphology and size were observed. Following the milling process, the mixed powder particles exhibited enhanced plastic deformation capabilities. The impact between the grinding balls, powder, and milling jar created a micro-forging effect, leading to the formation of flaky and fragmented powder particles. Through a combination of micro-forging and crushing actions, the particle size was progressively refined. The observed trend in particle size reduction with increasing milling time can be attributed to the dominance of the fracturing mechanism during the process [41]. As the powder is broken down, its particle size decreases. Under identical milling conditions, a smaller particle size typically corresponds to a higher oxygen content due to the increased specific surface area and surface energy, which enhance the powder’s ability to adsorb oxygen. Consequently, the powder milled for 8 h likely exhibited the highest oxidation level. Previous studies indicate that excessive oxygen content can result in oxide inclusions and the formation of a continuous oxide film on the powder particles’ surface [42]. This oxide layer impedes particle activation and bonding during sintering, leading to reduced density and deteriorating the mechanical properties of the sintered material. No mechanical alloying was observed in the powder milled for 8 h. Therefore, a shorter milling duration is recommended to mitigate the effects of oxidation, as confirmed by the scanned images in Figure 6. Powders milled for 0.5 and 1 h showed uneven mixing, while those milled for 2 h exhibited a more uniform blend, making 2 h the optimal milling time in this study for subsequent sintering.

### 3.2. Effect of Process Parameters on Properties

**Figure 7:**
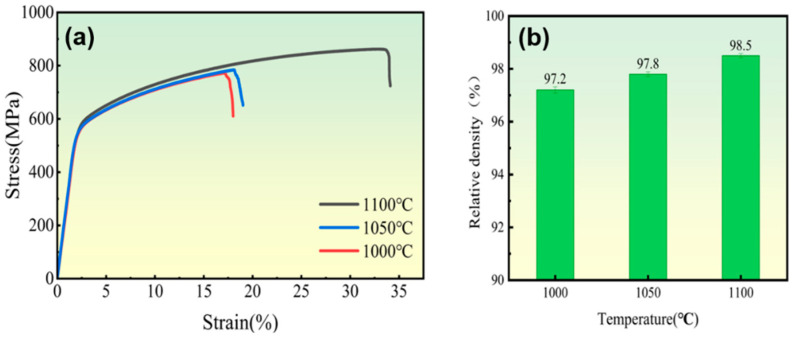
Stress–strain curves (**a**) and relative density measurements (**b**) of sintered Ni-17Mo alloys processed at various sintering temperatures, demonstrating the relationship between sintering conditions and mechanical performance.

Figure 7 presents the stress–strain curves and relative densities for the sintered Ni-17Mo alloy at various temperatures. Additionally, key mechanical parameters from Figure 7 are tabulated in Table 1 for analysis. The data reveal that the samples sintered at 1000 °C and 1050 °C exhibit similar tensile properties, while the sample sintered at 1100 °C demonstrates superior tensile performance, with a yield strength of 579.5 MPa, a tensile strength of 859.6 MPa, and an elongation of 31.6%. Compared to the lower sintering temperatures, both the tensile strength and elongation are notably improved at 1100 °C. This improvement suggests that a denser structure forms at 1100 °C due to enhanced thermal diffusion. Based on the tensile mechanical properties and relative density data across different sintering temperatures, it can be concluded that the optimal sintering temperature for the Ni-17Mo alloy is 1100 °C.

### 3.3. Effect of Adding Gr on Alloy Matrix

**Figure 8:**
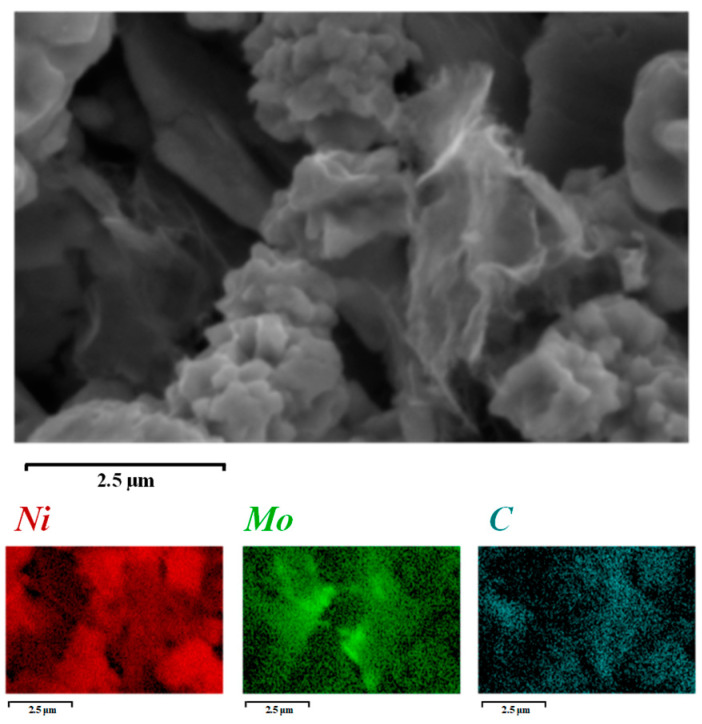
SEM image of 0.5 vol.% RGO/Ni-17Mo alloy composite powder and its corresponding EDS elemental mappings, revealing the dispersion of RGO within the matrix.

Figure 8 displays the SEM image of the RGO/Ni-17Mo composite powder after mixing, along with its corresponding EDS spectrum. The energy dispersive spectroscopy (EDS) analysis reveals that the alloy powder and RGO are uniformly blended, which supports the homogeneity of the composition in the resulting composite material. Additionally, the analysis illustrates the “brick-and-mortar” arrangement of the alloy flake powder and RGO, facilitating the formation of a distinct bionic layered structure within the composite [43,44,45].

**Figure 9:**
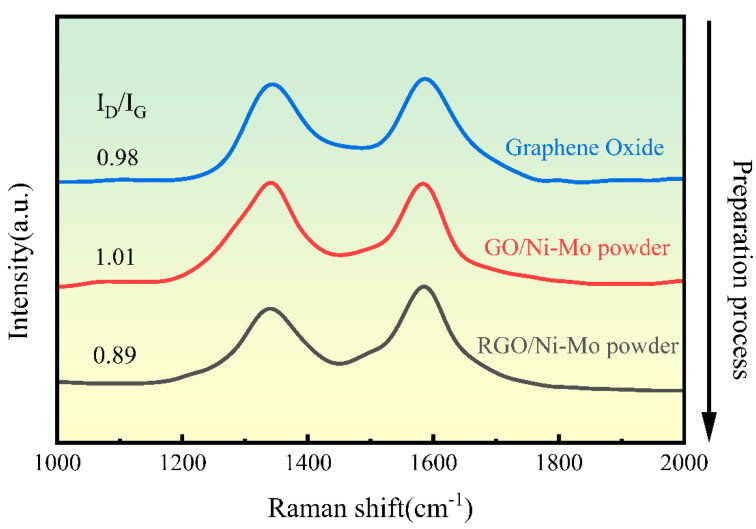
Raman spectra of GO powders at different stages of the preparation process, highlighting changes in defect structures.

Figure 9 presents the Raman spectra of the original, the ultrasonically dispersed, and the reduced GO powders obtained at different stages of the preparation process. Raman spectroscopy allows for monitoring the quality of Gr throughout the preparation process. The blue curve represents the Raman spectrum of the original GO powder, with an I_D_/I_G_ ratio (corresponding to the peak area ratio [46]) of 0.92. The red curve corresponds to the Raman spectrum of composite powder, showing an I_D_/I_G_ ratio of 1.03. This increase indicates further degradation of the Gr structure following the ultrasonic dispersion of GO. This structural damage is linked to a reduction in the diameter of GO sheets caused by the ultrasonic treatment, which leads to a higher volume fraction of Gr with incomplete edges, thus raising the I_D_/I_G_ ratio. The black curve shows the Raman spectrum after thermal reduction at 500 °C, with an I_D_/I_G_ ratio of 0.96. This result demonstrates that high-temperature thermal reduction effectively aids in repairing the Gr structure.

**Figure 10:**
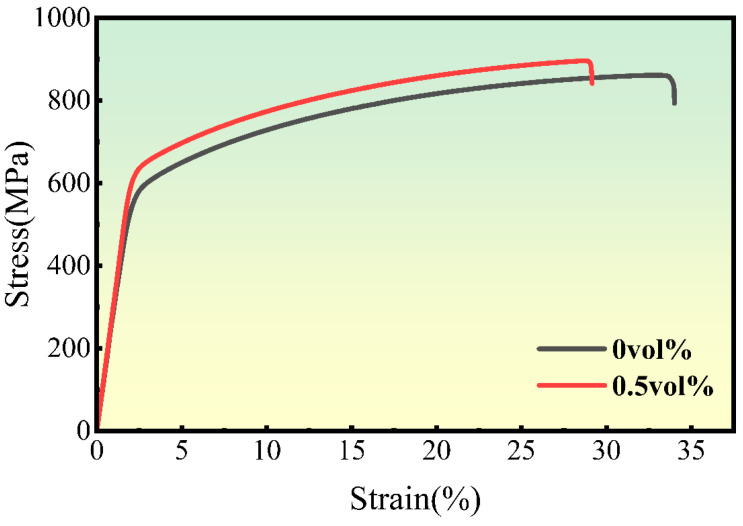
Stress–strain curves for Ni-17Mo alloy composites with different RGO contents, illustrating the influence of RGO on mechanical properties.

**Figure 11:**
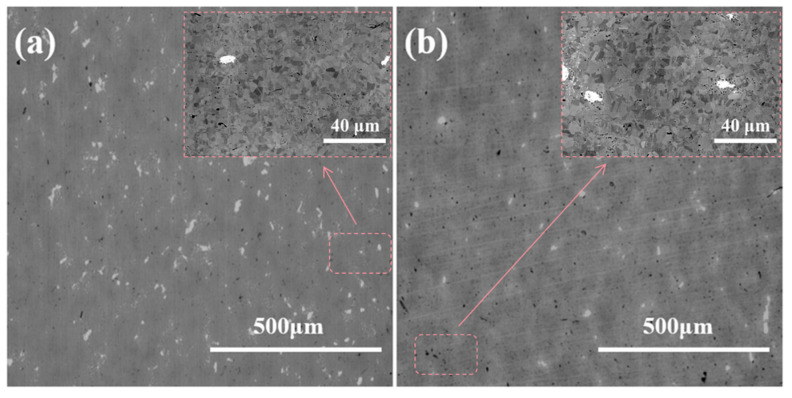
SEM images showing the microstructural variations in Ni-17Mo alloy composites with different RGO contents. (**a**) 0 vol% RGO. (**b**) 0.5 vol% RGO.

Figure 10 presents the room temperature tensile stress–strain curve of RGO/Ni-17Mo composite sintered bodies with varying RGO contents tested at a tensile rate of 10^−3^ mm/s. The sintered Ni-17Mo alloy exhibits a yield strength of 579.5 MPa, a tensile strength of 859.6 MPa, and an elongation at break of 31.6%. With the addition of 0.5 vol.% RGO, the yield and tensile strengths of the composite sintered body increase to 635.9 MPa and 894.3 MPa, respectively, although the elongation at break decreases to 26.4%. This enhancement in strength is attributed to the formation of Mo-rich carbides in the 0.5 vol% RGO/Ni-17Mo alloy composite. As seen in Figure 11, the introduction of RGO not only increases the irregular black contrast structures associated with cavities but also results in an increase in white regions. According to the literature [47], these white areas correspond to Mo-rich carbides. Owing to molybdenum’s strong affinity for carbon, the addition of RGO promotes carbide formation. Furthermore, RGO tends to agglomerate and stack, which increases the sample’s porosity and hinders matrix densification [48]. Consequently, while carbides enhance the composite’s strength and hardness, their presence also induces stress concentrations during tensile deformation, leading to premature fracture and reduced plasticity.

## 4. Conclusions

In summary, this study investigates the fabrication of Gr-nanosheet-reinforced Ni-17Mo alloy composites using a modified powder metallurgy approach. The effects of milling time on powder uniformity and particle size were examined, and optimal milling conditions were determined for subsequent sintering at temperatures of 1000, 1050, and 1100 °C. Mechanical properties of the sintered samples were tested, and composites were created by incorporating 0.5 vol.% RGO. XRD and SEM analyses revealed that milling time affected the alloy powder’s grain size and morphology, with the optimum milling duration identified as 2 h to achieve uniform mixing. The sintering temperature significantly influenced the mechanical properties, with samples sintered at 1100 °C showing the best performance, including improved yield strength (579.5 MPa), tensile strength (859.6 MPa), and elongation (31.6%). The addition of 0.5 vol.% RGO to the Ni-17Mo alloy improved strength, reaching 635.9 MPa yield strength and 894.3 MPa tensile strength, though elongation decreased to 26.4%. This enhancement was attributed to the formation of Mo-rich carbides, which strengthened the composite but also led to reduced plasticity due to stress concentration. The Raman spectroscopy results indicated that the Gr structure was slightly degraded during ultrasonic dispersion but was largely repaired by thermal reduction, confirming the importance of processing conditions on the final composite structure. These findings suggest that optimizing milling and sintering parameters is crucial for producing high-performance Gr/Ni-17Mo composites for potential applications in advanced nuclear reactors.

## Figures and Tables

**Table 1:** Statistical summary of mechanical testing results.

Samples	Yield Strength (MPa)	Ultimate Tensile Strength (MPa)	Elongation (%)	Hardness (HV)
1000 °C	544.3 ± 7.4	771.6 ± 5.5	15.9 ± 2.0	259 ± 9
1050 °C	557.2 ± 9.2	784.9 ± 15.2	16.7 ± 3.1	246 ± 7
1100 °C	579.5 ± 4.5	859.6 ± 8.3	31.6 ± 2.5	190 ± 3

## Data Availability

The original contributions presented in this study are included in the article. Further inquiries can be directed to the corresponding author.
